# Elasticity Values as a Predictive Modality for Response to Neoadjuvant Chemotherapy in Breast Cancer

**DOI:** 10.3390/cancers16020377

**Published:** 2024-01-16

**Authors:** Min Ji Kim, Na Lae Eun, Sung Gwe Ahn, Jee Hung Kim, Ji Hyun Youk, Eun Ju Son, Joon Jeong, Yoon Jin Cha, Soong June Bae

**Affiliations:** 1 Department of Surgery, Gangnam Severance Hospital, Yonsei University College of Medicine, Seoul 06273, Republic of Korea; minji1766@yuhs.ac (M.J.K.); asg2004@yuhs.ac (S.G.A.); gsjjoon@yuhs.ac (J.J.); 2 Institute for Breast Cancer Precision Medicine, Yonsei University College of Medicine, Seoul 06273, Republic of Korea; ok8504@yuhs.ac; 3 Department of Radiology, Gangnam Severance Hospital, Yonsei University College of Medicine, Seoul 06273, Republic of Korea; enrlove@yuhs.ac (N.L.E.); jhyouk@yuhs.ac (J.H.Y.); ejsonrd@yuhs.ac (E.J.S.); 4 Division of Medical Oncology, Department of Internal Medicine, Gangnam Severance Hospital, Yonsei University College of Medicine, Seoul 06273, Republic of Korea; 5 Department of Pathology, Gangnam Severance Hospital, Yonsei University College of Medicine, Seoul 06273, Republic of Korea

**Keywords:** breast neoplasm, neoadjuvant therapy, ultrasound, elasticity imaging techniques, treatment response

## Abstract

**Simple Summary:**

Although shear-wave elastography has been utilized in diagnosing a malignant breast lesion and axillary lymph node metastasis, its potential role in predicting treatment response to neoadjuvant chemotherapy has not been thoroughly explored. In this study, we aimed to assess the possibility of elasticity values measured using SWE as a predictive marker for neoadjuvant chemotherapy in breast cancer. Our findings indicate that low tumor stiffness, as measured using SWE, was significantly associated with an excellent treatment response following neoadjuvant chemotherapy. This relationship was particularly evident in hormone-receptor-positive, HER2-negative breast cancer, and triple-negative breast cancer. Furthermore, we identified an inverse correlation between tumor stiffness and the tumor-infiltrating lymphocyte level, suggesting that tumors with high TIL levels tend to exhibit lower stiffness. Our findings suggest that SWE could be a useful tool in predicting treatment response and guiding treatment decisions in neoadjuvant chemotherapy for patients with breast cancer.

**Abstract:**

Shear-wave elastography (SWE) is an effective tool in discriminating malignant lesions of breast and axillary lymph node metastasis in patients with breast cancer. However, the association between the baseline elasticity value of breast cancer and the treatment response of neoadjuvant chemotherapy is yet to be elucidated. Baseline SWE measured mean stiffness (E-mean) and maximum stiffness (E-max) in 830 patients who underwent neoadjuvant chemotherapy and surgery from January 2012 to December 2022. Association of elasticity values with breast pCR (defined as ypTis/T0), pCR (defined as ypTis/T0, N0), and tumor-infiltrating lymphocytes (TILs) was analyzed. Of 830 patients, 356 (42.9%) achieved breast pCR, and 324 (39.0%) achieved pCR. The patients with low elasticity values had higher breast pCR and pCR rates than those with high elasticity values. A low E-mean (adjusted odds ratio (OR): 0.620; 95% confidence interval (CI): 0.437 to 0.878; *p* = 0.007) and low E-max (adjusted OR: 0.701; 95% CI: 0.494 to 0.996; *p* = 0.047) were independent predictive factors for breast pCR. Low elasticity values were significantly correlated with high TILs. Pretreatment elasticity values measured using SWE were significantly associated with treatment response and inversely correlated with TILs, particularly in HR+HER2- breast cancer and TNBC.

## 1. Introduction

Neoadjuvant chemotherapy has gained widespread utilization in patients with breast cancer, aiming to downsize breast tumors, eradicate axillary lymph node metastasis, and enhance the likelihood of breast-conserving surgery [1,2]. Moreover, neoadjuvant chemotherapy could evaluate the clinical efficacy of novel drugs [3] and the treatment response determines the regimen of following adjuvant therapy [4,5]. The pathologic complete response (pCR) after neoadjuvant chemotherapy is well known to be associated with a favorable prognosis, especially in patients with HER2-positive (HER2+) breast cancer and triple-negative breast cancer (TNBC) [6,7,8,9]. Consequently, considerable research efforts have focused on identifying predictive biomarkers, including radiologic parameters, breast cancer subtypes, histologic grade, Ki-67, and tumor-infiltrating lymphocytes (TILs), to anticipate treatment response of neoadjuvant chemotherapy [10,11].

In clinical settings, various radiologic methods such as ultrasound and breast MRI are frequently employed to track the effectiveness of neoadjuvant chemotherapy. While breast MRI stands out as the most precise tool for gauging tumor response, there remains a concern about potential false-negative or false-positive outcomes. Additionally, the widespread applicability of breast MRI is limited, and its use is constrained in patients with specific conditions. Similarly, ultrasound, relying on grayscale images, does not fare well in accurately predicting the response to neoadjuvant chemotherapy. Consequently, there is still a lack of universally satisfactory imaging modalities for reliably forecasting the efficacy of neoadjuvant chemotherapy [12,13].

Shear-wave elastography (SWE) is a non-invasive imaging method that enables the reproducible quantification of tissue stiffness using ultrasound. In addition, SWE is easily incorporated into the standard workup for patients with breast cancer. This modality has proven to be effective in discriminating malignant breast lesions from benign or normal mammary tissues, as malignant lesions tend to exhibit higher stiffness [14]. Furthermore, in patients diagnosed with ductal carcinoma in situ, the combination of SWE with ultrasound examination has accurately predicted the upgrade to invasive cancer based on stiffness discrepancies [15]. In addition, the application of SWE in axillary evaluation can aid the prediction of tumor metastasis [16,17,18]. However, despite these significant strides, the impact of tumor stiffness on treatment response to neoadjuvant chemotherapy is not elucidated in breast cancer.

The present study aimed to assess whether the degree of tumor stiffness in breast cancers, measured using SWE before the treatment initiation, was associated with the response to neoadjuvant chemotherapy. Additionally, we explored the clinicopathologic features according to the elasticity values assessed using SWE.

## 2. Patients and Methods

### 2.1. Study Population

Our study was approved by the Institutional Review Board at Gangnam Severance Hospital, Yonsei University, Seoul, Republic of Korea (Local IRB no. 3-2020-0307), following the Good Clinical Practice guidelines and the Declaration of Helsinki. The need for informed consent was waived under the approval of the IRB due to its retrospective design.

**Figure 1:**
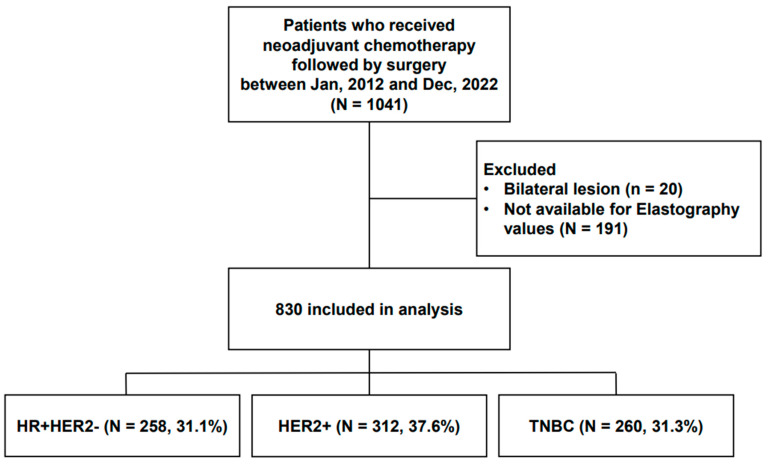
Study population.

As shown in Figure 1, between January 2012 and December 2022, 1041 patients received neoadjuvant chemotherapy followed by curative surgery. Of these, 830 patients were finally assessable and included in this study. The clinicopathologic data, including age at diagnosis of breast cancer, elasticity values measured using SWE, histologic grade (HG), estrogen receptor (ER), progesterone receptor (PR), human epidermal growth factor receptor 2 (HER2), clinical subtypes of breast cancer, Ki-67 index, TILs, pretreatment clinical T and clinical N stages, treatment regimen, and post-treatment pathologic T and pathologic N stages, were collected from the electronic medical records. The clinical stage was evaluated on preoperative radiologic modalities including mammography, ultrasound, and breast MRI per the 8th edition of the American Joint Committee on Cancer guidelines.

### 2.2. Pathologic Evaluation, Immunohistochemistry (IHC), and TIL Assessment

Pathologic evaluation and IHC interpretation were performed using light microscopy (BX53 upright microscope; Olympus, Tokyo, Japan). The HG of the breast tumor was determined based on the modified Scarf–Bloomer–Richardson grading system [19].

ER (clone 6F11; dilution of 1:200; Leica Biosystems, Wetzlar, Germany), PR (clone 16; dilution of 1:500; Leica Biosystems), and HER2 (clone 4B5; dilution of 1:5; Ventana Medical System, Oro Valley, AZ, USA) staining was performed according to the guideline of the 2018 American Society of Clinical Oncology/College of American Society of Clinical Oncology/College of American Pathologists [20,21]. For the estrogen receptor (ER) and progesterone receptor (PR), nuclear staining values of 1% or higher were considered positive. Cases with strong and circumferential membranous HER2 immunoreactivity (3+) were considered positive, while those with 0 and 1+ HER2 staining were considered negative. Samples with equivocal HER2 expression (2+) underwent further evaluation for HER2 gene amplification via silver in situ hybridization (SISH).

In all patients, ultrasound-guided core needle biopsy with a 14-gauge semiautomated biopsy gun was performed under local anesthesia. At least three core samples were obtained per patient. Core needle biopsy samples were classified into the following subtypes:

λ Hormone-receptor-positive, HER2-negative (HR+HER2-): ER-positive and/or PR-positive, and HER2-negative.
λ HER2+: HER2-positive regardless of ER and PR status.
λ Triple-negative breast cancer (TNBC): ER-negative, PR-negative, and HER2-negative.

The TIL levels were concurrently assessed following the International TIL Working Group guidelines [22]. In core needle biopsy samples, all cores containing invasive tumor cells were evaluated. Apart from polymorphonuclear leukocytes, other mononuclear cells, including lymphocytes and plasma cells, were counted, and the average score was reported as a percentage [23]. For a statistical analysis, a 30% cutoff was applied to categorize patients into low-TIL (<30%) and high-TIL (≥30%) groups [24].

### 2.3. Elastography

Four radiologists, each with 5–10 years of experience, conducted all breast ultrasound examinations with SWE. In essence, we utilized the Aixplorer ultrasound system (SuperSonic Imagine, Aix-en-Provence, France), along with its ShearWave™ elastography mode. This mode was equipped with a 4–15 MHz linear-array transducer to capture SWE images of breast lesions before neoadjuvant chemotherapy. During the examinations, we configured a rectangular field-of-view box to encompass both the breast lesion and the surrounding normal tissue. This setting revealed a semitransparent color map illustrating tissue stiffness overlaid on the gray-scale image. The color range extended from dark blue, indicating the lowest stiffness, to red, indicating the highest stiffness (0–300 kPa). The transducer was held steady for a few seconds to allow the SWE image to stabilize. To minimize artifacts, the probe lightly touched the skin without consciously applying any vibration or compression. In addition, generous amounts of contact jelly were used, and the patient was instructed to hold their breath. SWE images were captured at least twice, and the most representative image with sufficient quality and the fewest artifacts was chosen and saved. Elasticity values, such as mean stiffness (E-mean) and maximum stiffness (E-max), were automatically computed by placing a fixed 20mm circular region of interest (ROI) on the stiffest part of the breast lesion, including the peritumoral areas [14].

We used receiver operating characteristic (ROC) curve analyses to evaluate the sensitivities and specificities of elasticity values for predicting breast pCR after neoadjuvant chemotherapy. The optimal cut-off points of elasticity values that maximize sensibility and specificity were established. Different subtypes of breast cancer respond differently to neoadjuvant chemotherapy, and there was a statistical difference in elasticity values among the subtypes (Table 1); we established the cut-off values of elasticity values for the entire cohort and each breast cancer subtype separately. The ideal points to classify a high or low E-mean, and E-max, were as follows: 190.75 and 221.35 in the entire cohort; 179.7 and 192.9 in HR+HER2- breast cancer; 119.2 and 145.8 in HER2+ breast cancer; 190.35 and 218 in TNBC, respectively.

### 2.4. Statistical Analysis

We compared continuous variables using Student’s *t*-test and categorical variables using either the chi-square test or Fisher’s exact test. The pathologic complete response (pCR) was defined as the absence of invasive cancer cells in both the breast and axillary lymph nodes (ypT0/is, ypN0), while breast pCR was specifically defined as the absence of invasive cancer cells in the breast (ypT0/is). These criteria were based on the pathologic evaluation of the surgical specimen after neoadjuvant chemotherapy [25]. To identify significant factors related to pCR and breast pCR, we conducted univariable and multivariable logistic regression analyses. Additionally, we explored the relationship between the elasticity values and TIL level using Pearson’s Correlation test. All analyses were performed using SPSS version 25 (SPSS; Chicago, IL, USA). A *p*-value less than 0.05, two-sided, was considered statistically significant, and 95% confidence intervals (CIs) not including 1 were deemed significant as well.

## 3. Results

### 3.1. Baseline Characteristics

The median age of the patients included in this analysis (N = 830) was 48 years (range, 21–80). Of these, 31.8% were clinical T3, and 84.5% were clinical node-positive. Among the whole cohort, 258 (31.1%) had HR+HER2- breast cancer, 312 (37.6%) had HER2+ breast cancer, and 260 (31.3%) had TNBC (Table S1). The majority (98.6%) of patients with HER2+ breast cancer received neoadjuvant chemotherapy with HER2-targeted therapy: 295 of 312 (94.6%) patients received docetaxel, carboplatin, trastuzumab, and pertuzumab (TCHP); 14 of 312 (4.5%) patients received adriamycin, and cyclophosphamide followed by taxane with trastuzumab; 3 of 312 (0.9%) patients received anthracycline and taxane-based chemotherapy without HER2-targeted therapy. All patients with HER2-negative breast cancer received adriamycin- and taxane-based neoadjuvant chemotherapy. The average values of the E-mean and E-max were significantly higher in patients with HR+HER2- breast cancer than the rest of the patients (E-mean = 188.47 kPa, *p* = 0.036; E-max = 216.1 7kPa, *p* = 0.022, respectively).

According to the cut-off points of elasticity values, the proportion of a low E-mean and low E-max was as follows: 55.3% (459 of 830) and 56.7% (471 of 830) in all patients (Table S1), 44.2% (114 of 258) and 42.2% (109 of 258) in HR+HER2- breast cancer (Table S2), 60 of 312 (19.2%) and 70 of 312 (22.4%) in HER2+ breast cancer (Table S3), and 146 (56.2%) of 260 and 151 (58.1%) of 260 in TNBC (Table S4), respectively. In HER2+ breast cancer, tumors with low elasticity values showed as higher than those with high elasticity values. (Table S3). In HR+HER2- breast cancer and TNBC, an inverse correlation between elasticity values and TILs was observed (Tables S2 and S4).

### 3.2. Relationship between the Elasticity Values and TIL Level

**Figure 2:**
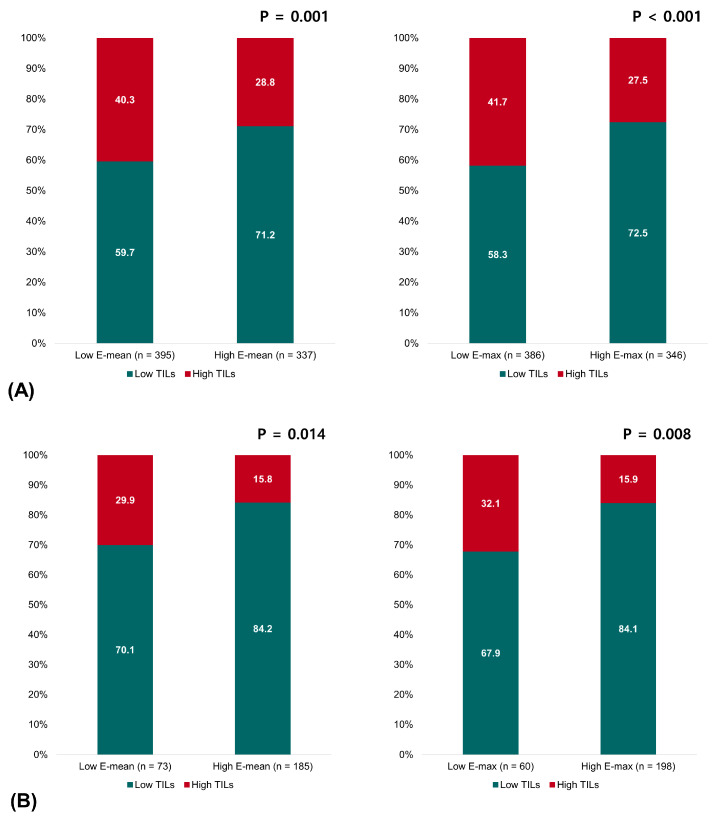

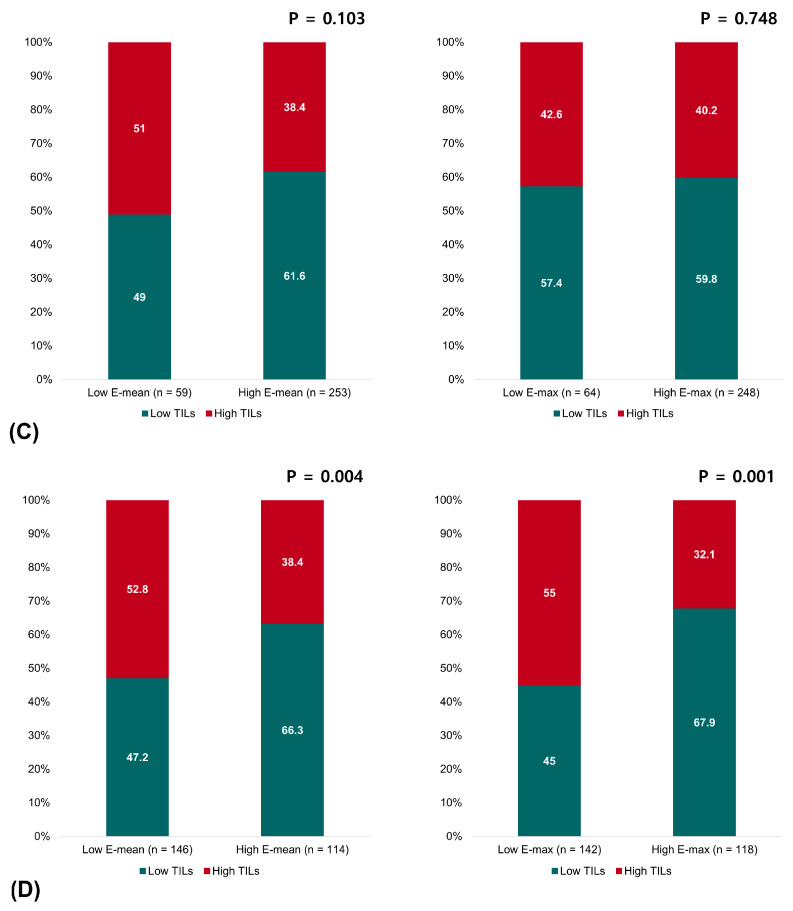
Relationship between the elasticity values and TILs in (**A**) all patients, (**B**) patients with HR+HER2- breast cancer, (**C**) patients with HER2+ breast cancer, and (**D**) patients with triple-negative breast cancer.

Pretreatment TILs on biopsy samples were evaluated in 732 of 830 (88.2%) patients. Overall, tumors with high TILs significantly predominated with low elasticity values: 40.3% in the low-E-mean group vs. 28.8% in the high-E-mean group (*p* = 0.001) and 41.0% in the low-E-max group vs. 27.4% in the high-E-max group (*p* < 0.001). Analyzing by breast cancer subtypes, we observed similar trends in the patients with HR+HER2- breast cancer and TNBC. In HR+HER2- breast cancer, tumors with high TILs were more commonly observed in low-E-mean and -E-max groups: 26.5% of the low-E-mean group vs. 14.7% of the high-E-mean group (*p* = 0.024) and 26.0% of the low-E-max group vs. 15.2% of the high-E-max group (*p* = 0.039). In TNBC, this inverse correlation between TILs and elasticity became even more apparent. High TILs were found in 52.8% of the low-E-mean group vs. 33.7% of the high-E-mean group (*p* = 0.004) and 53.1% of the low-E-max group vs. 32.7% of the high-E-max group (*p* = 0.002). In contrast, no significant relationship existed between the elasticity values and TILs in HER2+ breast cancer (Figure 2).

### 3.3. Pathologic Complete Response According to the Elasticity Values

Overall, 356 (42.9%) patients achieved the breast pCR, and 324 (39.0%) patients achieved the pCR after neoadjuvant chemotherapy (Table 2). In addition, the breast pCR rate and the pCR rate were highest in patients with HER2+ breast cancer (66.3% and 62.8%), followed by TNBC (48.5% and 45.0%), and HR+HER2- breast cancer (8.9% and 4.3%).

In all patients, pretreatment low elasticity values were associated with the response to neoadjuvant chemotherapy (Table 2). The breast pCR rate and pCR rate were 48.8% and 43.6% in the low-E-mean group vs. 35.6% and 33.4% in the high-E-mean group (*p* < 0.001 and *p* < 0.001, respectively) and 48.8% and 43.5% in the low-E-max group vs. 35.1% and 33.1% in the high-E-max group (*p* < 0.001 and *p* = 0.002, respectively). In the multivariable analysis, a low E-mean (adjusted OR: 0.620; 95% CI: 0.437 to 0.878; *p* = 0.007) and low E-max (adjusted OR: 0.701; 95% CI: 0.494 to 0.996; *p* = 0.047) still remained as independent factors for predicting the breast pCR, but not for the pCR (Table 3).

Although the high elasticity values were consistently related to the poor treatment response, the statistically significant elasticity values were different by the tumor subtypes (Table 2). The proportion of breast pCR and pCR was significantly higher in HR+HER2- breast cancer and TNBC with low elasticity values (both E-mean and E-max). Meanwhile, HER2+ breast cancer had a significantly different proportion of the breast pCR rate with the E-max alone. With the multivariable analysis, low elasticity (both E-mean and E-max) was an independent factor for breast pCR in HR+HER2- breast cancer (E-mean—adjusted OR: 0.333, 95% CI: 0.120–0.926, *p* = 0.035; E-max—adjusted OR: 0.322, 95% CI: 0.116–0.893, *p* = 0.030). Regarding TNBC, low elasticity (both E-mean and E-max) was significantly associated with both breast pCR (E-mean—adjusted OR: 0.394, 95% CI: 0.226–0.688, *p* = 0.001; E-max—adjusted OR: 0.512, 95% CI: 0.295–0.891, *p* = 0.018) and pCR (E-mean—adjusted OR: 0.440, 95% CI: 0.251–0.772, *p* = 0.004; E-max—adjusted OR: 0.570, 95% CI: 0.326–0.997; *p* = 0.049) (Table 3). The elasticity value had no significant effect on breast pCR or pCR of HER2+ breast cancer.

## 4. Discussion

Herein, we evaluated the relationship between the tumor stiffness represented using SWE and treatment response in patients with breast cancer who received neoadjuvant chemotherapy. Because the elasticity values (E-mean and E-max) used in this study were measured in the malignant breast lesion rather than in the axillary lymph node, the treatment response was evaluated in two aspects: the breast pCR and the pCR. Our findings revealed a significant link between elevated stiffness, as indicated using SWE measurements, and a diminished rate of breast pCR or pCR following neoadjuvant chemotherapy. It could be assumed that high elasticity values may be caused by extracellular matrix (ECM) stiffness. When invasive cancer cells penetrate the basement membrane into the adjacent stroma, which is called a desmoplastic reaction [26], it makes ECM denser, by activating the cancer-associated fibroblast and inducing connective fibers including tenascin and fibronectin [27,28]. Earlier research has described that ECM stiffness enhances cancer cell growth, progression, metastasis, and drug resistance [26,27,29].

The association between low elasticity values and a favorable response to neoadjuvant chemotherapy has been consistently represented [30,31,32,33,34,35]. In addition, several antecedent studies underscore the superiority of pretreatment SWE over ultrasound alone in predicting neoadjuvant chemotherapy response. Furthermore, an artificial intelligence model or nomogram developed by incorporating radiologic parameters from medical images including SWE and clinicopathologic data has exhibited excellent diagnostic predictive performance for the neoadjuvant chemotherapy response [32,33]. These results not only highlight the synergistic potential of various data sources but also emphasize the role of elasticity values as a pivotal factor in accurately predicting the response to neoadjuvant chemotherapy.

In contrast to previous studies lacking subtype stratification, our analysis with a large cohort provides substantial statistical power to assess whether the elasticity values were predictive for the treatment response stratified by breast cancer subtypes: we observed an overall better treatment response with low elasticity values, with significant predictive elasticity values varying for each subtype. Differences in elasticity values could be related to the TIL level of each tumor subtype. Over 80% of HR+HER2- breast cancer belonged to the low-TIL group, and their elasticity was higher than HER2+ or TNBC subtypes. In contrast to mesenchymal components or tumor cells that are tightly attached to cell junctions, TILs have no adhesive properties [36], which could explain the higher elasticity of HR+HER2- breast cancer.

Interestingly, the elasticity values were predictive factors for the treatment response in HR+HER2- breast cancer and TNBC, whereas the multivariable analysis showed an insignificant association between the elasticity values and treatment response in HER2+ breast cancer. The efficacy of dual HER2-targeted therapy likely counteracts the negative impact of high elasticity on chemotherapy efficacy. Meanwhile, Yuan et al. reported a significant correlation between the elasticity value (E-max) and neoadjuvant chemotherapy response in the HER2-enriched subtype. This discrepancy could be attributed to a difference in the number of patients and administration of HER2-targeted therapy. In our study, we included 312 patients with HER2+ breast cancer, with 94% of them receiving HER2-targeted therapy involving trastuzumab and pertuzumab. Conversely, the prior study encompassed only 26 patients with HER2+ breast cancer, and all of them exclusively received chemotherapy without HER2-targeted therapy.

Notably, recent reports demonstrated that early reduction in elasticity values is more accurate in predicting the treatment response than baseline SWE features [12,37]. In line with this, findings from the TBCR026 and PHERGAIN trials underscore the importance of early reduction in the standardized uptake value as a predictive marker for the response to neoadjuvant systemic therapy, particularly when HER2-targeted drugs are involved in the treatment of HER2+ breast cancer [38,39]. Accordingly, it becomes evident that the early reduction in elasticity values, rather than the baseline elasticity value, may carry more significance in predicting the treatment response for patients with breast cancer undergoing neoadjuvant chemotherapy, including those with HER2+ breast cancer receiving HER2-targeted therapy. The need for further studies is apparent to ascertain the most effective quantitative SWE parameters and to understand the impact of changes in elasticity values on the treatment response stratified by tumor subtypes.

Numerous studies have described the apparent relationship between high TILs and favorable clinical outcomes, especially in HER2+ breast cancer and TNBC [11,40]. Our study showed that TILs were inversely correlated with the elasticity values, especially in HR+HER2- breast cancer and TNBC. Similarly, a previous study showed that high elasticity values are significantly associated with low TILs in the adjuvant setting [36]. As a low elasticity value could imply high TIL infiltration, a favorable treatment response of TNBC could be explained by this context. However, for HR+HER- breast cancer, it is still intriguing that a low elasticity value predicted breast pCR, as this subtype usually has low TILs and poorly responds to neoadjuvant chemotherapy.

Our retrospective analysis has certain limitations. First, we evaluated the TILs from the core needle biopsy sample, which may not fully mirror the stromal TILs of the whole tumor. However, several studies reported that the TIL score in the core needle biopsy sample could be reliable, representing the TIL status of the entire tumor [23,41]. Second, although Ki-67 is a well-established predictive factor for pCR [42], this value is not routinely assessed in core needle biopsy samples at our institution. Because Ki-67 was evaluated in only about 26% of the entire cohort, it resulted in its exclusion from the multivariable analysis to maintain statistical power. Third, we separately established cut-off points for the entire cohort and each breast cancer subtype, considering the variations in the treatment regimens and response among different breast cancer subtypes. Accordingly, these cut-off points require validation in independent cohorts. Lastly, our study solely focused on the treatment response and did not assess the prognosis of patients after neoadjuvant chemotherapy. Extended follow-up studies are warranted to investigate the potential impact of elasticity values on post-neoadjuvant-chemotherapy survival.

## 5. Conclusions

In conclusion, our study revealed that the low elasticity values were significantly related to the better response to neoadjuvant chemotherapy and high TILs in patients with breast cancer. These findings were particularly evident in HR+HER2- breast cancer or TNBC. Our results propose SWE as a valuable adjunctive diagnostic tool for predicting the treatment response in patients with breast cancer undergoing neoadjuvant chemotherapy. Furthermore, this radiologic tool may have a potential role in representing the tumor-microenvironment status, further enhancing clinical relevance.

## Figures and Tables

**Table 1:** Baseline characteristics of patients according to breast cancer subtypes.

	HR+HER2- (*n* = 258)	HER2+ (*n* = 312)	TNBC (*n* = 260)	Total (*n* = 830)	*p*-Value
Age, median [range]	47 [29–78]	49 [31–78]	48 [21–80]	48 [21–80]	0.112
HG *, *n* (%)					<0.001
1 or 2	215 (91.1)	222 (82.5)	110 (47.4)	547 (74.2)	
3	21 (8.9)	47 (17.5)	122 (52.6)	190 (25.8)	
TILs *, *n* (%)					<0.001
<30%	191 (80.3)	159 (59.3)	126 (55.8)	476 (65.0)	
≥30%	47 (19.7)	109 (40.7)	100 (44.2)	256 (35.0)	
Clinical T stage, *n* (%)					<0.001
1 or 2	172 (66.7)	190 (60.9)	204 (78.5)	566 (68.2)	
3	86 (33.3)	122 (39.1)	56 (21.5)	264 (31.8)	
Clinical nodal status, *n* (%)					<0.001
negative	17 (6.6)	63 (20.2)	49 (18.8)	129 (15.5)	
positive	241 (93.4)	249 (79.8)	211 (81.2)	701 (84.5)	
Breast pCR					<0.001
Yes	23 (8.9)	207 (66.3)	126 (48.5)	356 (42.9)	
No	235 (91.1)	105 (33.7)	134 (51.5)	474 (57.1)	
pCR					<0.001
Yes	11 (4.3)	196 (62.8)	117 (45.0)	324 (39.0)	
No	247 (95.7)	116 (37.2)	143 (55.0)	506 (61.0)	
E-mean (mean, SD (kPa))	188.47 ± 66.55	174.76 ± 65.23	178.22 ± 61.99	180.11 ± 64.84	0.036
E-max (mean, SD (kPa))	216.17 ± 71.58	200.23 ± 71.93	203.60 ± 69.15	206.24 ± 71.20	0.022

* Missing values; HR, hormone receptor; HER2, human epidermal growth factor receptor 2; TNBC, triple-negative breast cancer; HG, histologic grade; TILs, tumor-infiltrating lymphocytes; E-max, maximum stiffness; E-mean, mean stiffness.

**Table 2:** pCR and breast pCR according to the elasticity values stratified by breast cancer subtypes.

			E-Mean			E-Max		
All Patients	Breast pCR	Total (*n* = 830)	Low (*n* = 459)	High (*n* = 371)	*p*-Value	Low (*n* = 471)	High (*n* = 359)	*p*-Value
	yes, *n* (%)	356 (42.9)	224 (48.8)	132 (35.6)	<0.001	230 (48.8)	126 (35.1)	<0.001
	no, *n* (%)	474 (57.1)	235 (51.2)	239 (64.4)		241 (51.2)	233 (64.9)	
**HR+HER2-**	**Breast pCR**	**Total** **(*n* = 258)**	**Low** **(*n* = 114)**	**High** **(*n* = 144)**	***p*-Value**	**Low** **(*n* = 109)**	**High** **(*n* = 149)**	***p*-Value**
	yes, *n* (%)	23 (8.9)	17 (14.9)	6 (4.2)	0.003	17 (18.3)	6 (4.0)	0.001
	no, *n* (%)	235 (91.1)	97 (85.1)	138 (95.8)		92 (81.7)	143 (96.0)	
**HER2+**	**Breast pCR**	**Total** **(*n* = 312)**	**Low** **(*n* = 60)**	**High** **(*n* = 252)**	***p*-Value**	**Low** **(*n* = 70)**	**High** **(*n* = 242)**	***p*-Value**
	yes, *n* (%)	207 (66.3)	46 (76.7)	161 (63.9)	0.060	54 (77.1)	153 (63.2)	0.030
	no, *n* (%)	105 (33.7)	14 (23.3)	91 (36.1)		16 (22.9)	89 (36.8)	
**TNBC**	**Breast pCR**	**Total** **(*n* = 260)**	**Low** **(*n* = 146)**	**High** **(*n* = 114)**	***p*-Value**	**Low** **(*n* = 151)**	**High** **(*n* = 109)**	***p*-Value**
	yes, *n* (%)	126 (48.5)	84 (57.5)	42 (36.8)	0.001	83 (55.0)	43 (39.4)	0.013
	no, *n* (%)	134 (51.5)	62 (42.5)	72 (63.2)		68 (45.0)	66 (60.6)	
			**E-mean**			**E-max**		
**All Patients**	**pCR**	**Total** **(*n* = 830)**	**Low** **(*n* = 459)**	**High** **(*n* = 371)**	***p*-Value**	**Low** **(*n* = 471)**	**High** **(*n* = 359)**	***p*-Value**
	yes, *n* (%)	324 (39.0)	200 (43.6)	124 (33.4)	0.003	205 (43.5)	119 (33.1)	0.002
	no, *n* (%)	506 (61.0)	259 (56.4)	247 (66.6)		266 (56.5)	240 (66.9)	
**HR+HER2-**	**pCR**	**Total (*n* = 258)**	**Low** **(*n* = 114)**	**High** **(*n* = 144)**	***p*-Value**	**Low** **(*n* = 109)**	**High** **(*n* = 149)**	***p*-Value**
	yes, *n* (%)	11 (4.3)	8 (7.0)	3 (2.1)	0.065 *	8 (7.3)	3 (2.0)	0.058 *
	no, *n* (%)	247 (95.7)	106 (93.0)	141 (97.9)		101 (92.7)	146 (98.0)	
**HER2+**	**pCR**	**Total** **(*n* = 312)**	**Low** **(*n* = 60)**	**High** **(*n* = 252)**	***p*-Value**	**Low** **(*n* = 70)**	**High** **(*n* = 242)**	***p*-Value**
	yes, *n* (%)	196 (62.8)	42 (70.0)	154 (61.1)	0.200	49 (70.0)	147 (60.7)	0.158
	no, *n* (%)	116 (37.2)	18 (30.0)	98 (38.9)		21 (30.0)	95 (39.3)	
**TNBC**	**pCR**	**Total** **(*n* = 260)**	**Low** **(*n* = 146)**	**High** **(*n* = 114)**	***p*-Value**	**Low** **(*n* = 151)**	**High** **(*n* = 109)**	***p*-Value**
	yes, *n* (%)	117 (45.0)	78 (53.4)	39 (34.2)	0.002	76 (50.3)	41 (37.6)	0.042
	no, *n* (%)	143 (55.0)	68 (46.6)	75 (65.8)		75 (49.7)	68 (62.4)	

* *p*-Values are obtained with Fisher’s exact test. pCR, pathologic complete response; E-max, maximum stiffness; E-mean, mean stiffness; HR, hormone receptor; HER2, human epidermal growth factor receptor 2; TNBC, triple-negative breast cancer.

**Table 3:** Odds ratio and 95% confidence interval of the elasticity values on pCR and breast pCR.

Breast pCR	All Patients		HR+HER2-		HER2+		TNBC	
	Odds Ratio (95% CI) *	*p*-Value *	Odds Ratio (95% CI) ^†^	*p*-Value ^†^	Odds Ratio (95% CI) ^†^	*p*-Value ^†^	Odds Ratio (95% CI) ^†^	*p*-Value ^†^
E-mean								
low	Ref		Ref		Ref		Ref	
high	0.620 (0.437–0.878)	0.007	0.333 (0.120–0.926)	0.035	0.585 (0.282–1.216)	0.151	0.394 (0.226–0.688)	0.001
E-max								
low	Ref		Ref		Ref		Ref	
high	0.701 (0.494−0.996)	0.047	0.322 (0.116−0.893)	0.030	0.554 (0.279−1.101)	0.092	0.512 (0.295−0.891)	0.018
**pCR**	**All Patients**		**HR+HER2-**		**HER2+**		**TNBC**	
	Odds Ratio (95% CI) *	***p*** -Value *	**Odds Ratio (95% CI) ^†^**	***p*** **-Value ^†^**	**Odds Ratio (95% CI) ^†^**	***p*** **-Value ^†^**	**Odds Ratio (95% CI) ^†^**	***p*** **-Value ^†^**
E-mean								
low	Ref		Ref		Ref		Ref	
high	0.733 (0.512−1.051)	0.091	0.423 (0.099−1.800)	0.244	0.762 (0.381−1.525)	0.589	0.440 (0.251−0.772)	0.004
E-max								
low	Ref		Ref		Ref		Ref	
high	0.827 (0.575−1.189)	0.305	0.392 (0.092−1.675)	0.607	0.688 (0.357−1.324)	0.262	0.570 (0.326−0.997)	0.049

* Covariates for multivariable models were age (continuous value), clinical T stage (1 or 2 vs. 3), clinical N stage (positive vs. negative), histologic grade (I–II vs. III), TILs (<30% vs. ≥30%), and subtypes (HR+HER2- vs. HER2+ vs. TNBC). ^†^ Covariates for multivariable models were age (continuous value), clinical T stage (1 or 2 vs. 3), clinical N stage (positive vs. negative), histologic grade (I–II vs. III), and TILs (<30% vs. ≥30%). pCR, pathologic complete response; HR, hormone receptor; HER2, human epidermal growth factor receptor 2; TNBC, triple-negative breast cancer; E-max, maximum stiffness; E-mean, mean stiffness.

## Data Availability

The datasets generated and analyzed during the current study are available from the corresponding author on request.

## Supplementary Materials

The following supporting information can be downloaded at: https://www.mdpi.com/article/10.3390/cancers16020377/s1, Table S1: Baseline characteristics of patients according to elasticity values in all patients; Table S2. Baseline characteristics of patients according to elasticity values in HR+HER2- breast cancer; Table S3. Baseline characteristics of patients according to elasticity values in HER2+ breast cancer; Table S4. Baseline characteristics of patients according to elasticity values in triple-negative breast cancer.
