# Supplementary material for: Elasticity Values as a Predictive Modality for Response to Neoadjuvant Chemotherapy in Breast Cancer
Source: Cancers (Basel). 2024 Jan 16;16(2):377. doi: 10.3390/cancers16020377 (PMC10814692; doi:10.3390/cancers16020377)
Supplement: Supplementary file 1 [file cancers-16-00377-s001.zip › cancers-2817445-supplementary.pdf]

Table S1. Baseline characteristics of patients according to elasticity values in all patients.

	E-mean			E-max		
	Low (N = 459)	High (N = 371)	P-value	Low (N = 471)	High (N = 359)	P-value
Age, median [range]	49 [21-80]	48 [29-78]	0.523	49 [21-80]	48 [29-78]	0.118
HG*, n (%)			0.486*			0.586†
1 or 2	301 (75.3)	246 (73.0)		309 (75.0)	238 (73.2)	
3	99 (24.8)	91 (27.0)		103 (25.0)	87 (26.8)	
TILs*, n (%)			0.001*			<0.001†
< 30%	236 (59.7)	240 (71.2)		240 (59.0)	236 (72.6)	
≥ 30%	159 (40.3)	97 (28.8)		167 (41.0)	89 (27.4)	
Clinical T stage, n (%)			0.881			0.977
1 or 2	314 (68.4)	252 (67.9)		321 (68.2)	245 (68.2)	
3	145 (31.6)	119 (32.1)		150 (31.8)	114 (31.8)	
Clinical nodal status, n (%)			0.275			0.262
negative	77 (16.8)	52 (14.0)		79 (16.8)	50 (15.5)	
positive	382 (83.2)	319 (86.0)		392 (83.2)	392 (84.5)	

*Missing values

†P-values are obtained with Fisher's exact test.

HR, hormone receptor; HER2, human epidermal growth factor receptor 2; E-max, maximum stiffness; E-mean, mean stiffness; HG, histologic grade; TILs, tumor-infiltrating lymphocyte

Table S2. Baseline characteristics of patients according to elasticity values in HR+HER2- breast cancer.

	E-mean			E-max		
	Low (N = 114)	High (N = 144)	P-value	Low (N = 109)	High (N = 149)	P-value
Age, median [range]	48 [31-78]	47 [29-78]	0.287	48 [31-78]	47 [29-75]	0.261
HG*, n (%)			0.352*			0.310†
1 or 2	90 (89.1)	125 (92.6)		88 (88.9)	127 (92.7)	
3	11 (10.9)	10 (7.4)		11 (11.1)	10 (7.3)	
TILs*, n (%)			0.024*			0.039†
< 30%	75 (73.5)	116 (85.3)		74 (74.0)	117 (84.8)	
≥ 30%	27 (26.5)	20 (14.7)		26 (26.0)	21 (15.2)	
Clinical T stage, n (%)			0.111			0.090
1 or 2	82 (71.9)	90 (62.5)		79 (72.5)	93 (66.7)	
3	32 (28.1)	54 (37.5)		30 (27.5)	56 (33.3)	
Clinical nodal status, n (%)			0.312			0.678
negative	10 (8.8)	7 (4.9)		8 (7.3)	9 (6.0)	
positive	104 (91.2)	137 (95.1)		101 (92.7)	140 (94.0)	

*Missing values

†P-values are obtained with Fisher's exact test.

HR, hormone receptor; HER2, human epidermal growth factor receptor 2; E-max, maximum stiffness; E-mean, mean stiffness; HG, histologic grade; TILs, tumor-infiltrating lymphocyte

Table S3. Baseline characteristics of patients according to elasticity values in HER2+ breast cancer.

	E-mean			E-max		
	Low (N = 60)	High (N = 252)	P-value	Low (N = 70)	High (N = 242)	P-value
Age, median [range]	50 [32-73]	49 [31-78]	0.605	50 [32-73]	49 [31-78]	0.622
HG*, n (%)			0.051			0.095
1 or 2	46 (92.0)	176 (80.4)		53 (89.8)	169 (80.5)	
3	4 (8.0)	43 (19.6)		6 (10.2)	41 (19.5)	
TILs*, n (%)			0.137			0.549
< 30%	25 (50.0)	134 (61.5)		33 (55.9)	126 (60.3)	
≥ 30%	25 (50.0)	84 (38.5)		26 (44.1)	83 (39.7)	
Clinical T stage, n (%)			0.026			0.003
1 or 2	29 (48.3)	161 (63.9)		32 (45.7)	158 (65.3)	
3	31 (51.7)	91 (36.1)		38 (54.3)	84 (34.7)	
Clinical nodal status, n (%)			0.500			0.333
negative	14 (23.3)	49 (19.4)		17 (24.3)	46 (19.0)	
positive	46 (76.7)	203 (80.6)		53 (75.7)	196 (81.0)	

*Missing values

HER2, human epidermal growth factor receptor 2; E-max, maximum stiffness; E-mean, mean stiffness; HG, histologic grade; TILs, tumor-infiltrating lymphocytes

Table S4. Baseline characteristics of patients according to elasticity values in triple-negative breast cancer.

	E-mean			E-max		
	Low (N = 146)	High (N = 114)	P-value	Low (N = 151)	High (N = 109)	P-value
Age, median [range]	48 [21-80]	48 [29-74]	0.513	49 [21-76]	48 [29-80]	0.652
HG*, n (%)			0.718			0.435
1 or 2	63 (48.5)	47 (46.1)		66 (49.6)	44 (44.4)	
3	67 (51.5)	55 (53.9)		67 (50.4)	55 (55.6)	
TILs*, n (%)			0.004			0.002
< 30%	59 (47.2)	67 (66.3)		60 (46.9)	66 (67.3)	
≥ 30%	66 (52.8)	34 (33.7)		68 (53.1)	32 (32.7)	
Clinical T stage, n (%)			0.437			0.449
1 or 2	112 (76.7)	92 (80.7)		116 (76.8)	88 (80.7)	
3	34 (23.3)	22 (19.3)		35 (23.2)	21 (19.3)	
Clinical nodal status, n (%)			0.261			0.266
negative	24 (16.4)	25 (21.9)		25 (16.6)	24 (22.0)	
positive	122 (83.6)	89 (78.1)		126 (83.4)	85 (78.0)	

*Missing values

E-max, maximum stiffness; E-mean, mean stiffness; HG, histologic grade; TILs, tumor-infiltrating lymphocytes
